# Direct Observation of Unstained Biological Specimens in Water by the Frequency Transmission Electric-Field Method Using SEM

**DOI:** 10.1371/journal.pone.0092780

**Published:** 2014-03-20

**Authors:** Toshihiko Ogura

**Affiliations:** Biomedical Research Institute, National Institute of Advanced Industrial Science and Technology (AIST), Umezono, Tsukuba, Ibaraki, Japan; Dalhousie University, Canada

## Abstract

Scanning electron microscopy (SEM) is a powerful tool for the direct visualization of biological specimens at nanometre-scale resolution. However, images of unstained specimens in water using an atmospheric holder exhibit very poor contrast and heavy radiation damage. Here, we present a new form of microscopy, the frequency transmission electric-field (FTE) method using SEM, that offers low radiation damage and high-contrast observation of unstained biological samples in water. The wet biological specimens are enclosed in two silicon nitride (SiN) films. The metal-coated SiN film is irradiated using a focused modulation electron beam (EB) at a low-accelerating voltage. A measurement terminal under the sample holder detects the electric-field frequency signal, which contains structural information relating to the biological specimens. Our results in very little radiation damage to the sample, and the observation image is similar to the transmission image, depending on the sample volume. Our developed method can easily be utilized for the observation of various biological specimens in water.

## Introduction

Scanning electron microscopy (SEM) is an important technique for producing high-resolution images of biological samples [1]–[3]. To allow observation under high vacuum conditions and to avoid electrical radiation damage by SEM, the biological specimens must be prepared using glutaraldehyde fixation, negative staining, a cryo technique, and/or a metal coating [4]–[6]. These preparations also have positive effects in terms of contrast enhancement and allowing the biological specimens to remain uncharged. Until now, atmospheric and/or wet biological specimens have been observed using atmospheric holders [7]–[10]. However, using these methods, the specimens undergo heavy radiation damage caused by the electron beam (EB) [11]–[13], while unstained samples give a very poor contrast [9], [10]. Therefore, these systems further require glutaraldehyde fixation with negative staining or use of gold labels [9], [10]. In transmission electron microscopy (TEM) based system, the biological molecules in a water layer were observed by the environmental sample holder equipped to phase-plate TEM [14]. This system is effective to analyse the biological molecules in water layer.

Recently, we reported SEM-based methods for the analysis of biological specimens with low radiation damage [15]–[18]. These methods enable high-contrast imaging of atmospheric and/or wet unstained biological samples [15], [16]. The high contrast is accomplished through the secondary electron and/or soft X-ray generated from the metal-coated silicon nitride (SiN) thin film. The irradiated electrons are almost scattered and absorbed in the metal-coated thin film, which results in low radiation damage to the unstained biological samples. However, it is difficult to observe specimens in a water layer with a thickness greater than 10 μm using these methods.

Here, we present a newly developed imaging method resulting in no damage to unstained biological specimens in water, on the basis of a frequency transmission electric-field (FTE) system using SEM. Wet biological specimens are enclosed in two SiN films: the upper SiN film is coated with tungsten (W) and nickel (Ni) layers with a thickness of 20 and 5 nm, respectively. The W-Ni-coated SiN film is irradiated using a focused modulation EB at a frequency of 30−60 kHz and a low-accelerating voltage. Irradiated electrons are almost absorbed in the W layer; thus, the negative electric-field potential arises at this position. This negative potential is oscillated at the EB modulation frequency of 30−60 kHz. The electric-field oscillation is transmitted to the lower SiN film through the biological samples in water. Finally, a measurement terminal under the SiN film detects the electric frequency signal, providing structural information relating to the biological specimens. In our method, the biological samples are not directly irradiated via the EB; thus, electron radiation damage is completely avoided.

## Results

**Figure 1:**
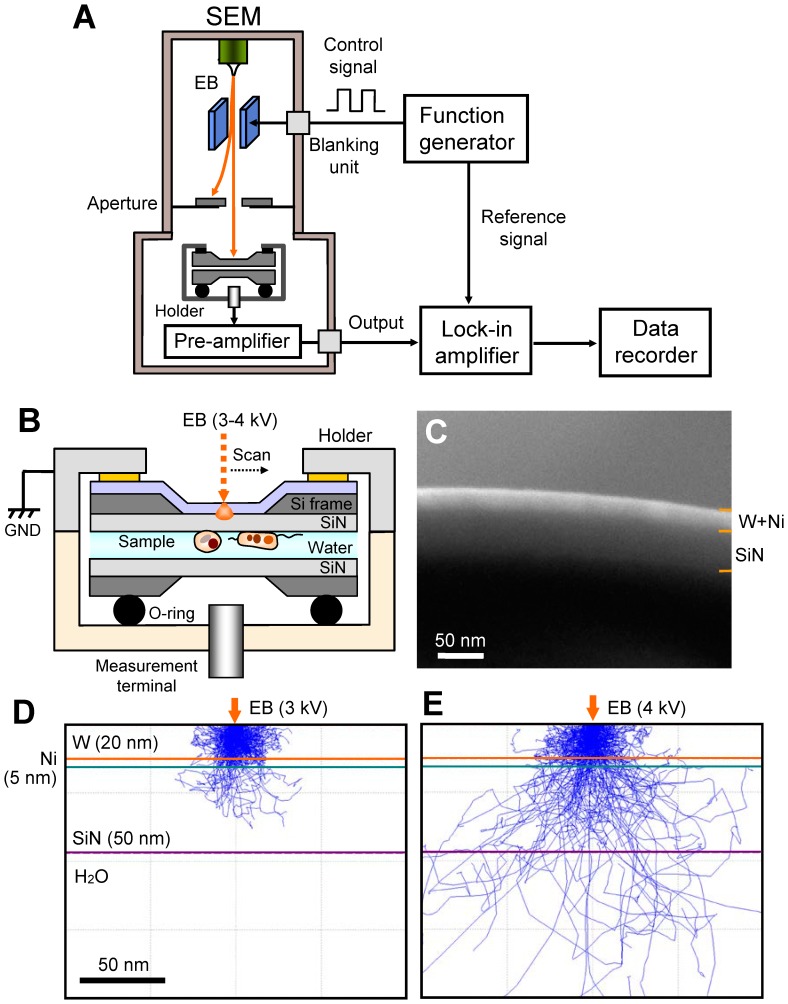
Experimental set-up and data acquisition system. (A) FTE imaging system. The scanning EB irradiates the upper side of the W–Ni-coated SiN film, which is modulated by the beam-blanking unit using a function generator at 30−60 kHz. The output signal from the lock-in amplifier is recorded by a data recorder. (B) Schematic of the atmospheric sample holder. The two SiN films with the liquid sample are sealed by the two sample-holding parts with double-sided tape, and the holding pieces are coupled using screws. The sample holder consists of an upper aluminium part and a lower acrylic resin. The wet biological specimens are enclosed in two SiN films: the upper side of the SiN film is coated with a W and Ni layer and connected to the system GND. (C) SEM cross-sectional image of a metal-coated SiN film consisting of a 20-nm-thick W layer, 5-nm Ni layer, and 50-nm SiN film. (D) MC simulation of the electron trajectories in the W–Ni-coated SiN film at 3-kV EB. The irradiated electrons are completely scattered and absorbed in the film. (E) MC simulation of 4-kV EB. A few electrons reach the water layer. Scale bars (C) and (D) 50 nm.

A schematic of our FTE imaging method using SEM is shown in Figure 1A. The electrostatic deflection system is installed in a thermionic SEM. A control, square wave, signal of 30−60 kHz from a function generator is applied to the deflection plate, producing the chopped EB. The metal-coated SiN film on the sample holder is irradiated using an EB with a low-accelerating voltage of 3−4 kV. A measurement terminal under the sample holder detects the transmission of an electric frequency signal to the biological specimens in water. The detection signal is output to a lock-in amplifier after pre-amplification. After the experimental procedure, FTE images are constructed using the detection signal and scanning signal of the EB from a data recorder.

Unstained and unfixed biological specimens in water are deposited under a metal-coated SiN film and sealed using a further 50-nm-thick SiN film, which is installed in the sample holder (Figure 1B). The sample holder consists of an upper aluminium part and a lower acrylic resin part. The upper part is connected to the system ground (GND); consequently, the metal layer on the SiN film is conducted to the GND. The lower, acrylic resin, part of the holder has a high resistivity; hence, measurement at the lower terminal of the holder is insulated from the metal-coated SiN film. This prevents direct detection of the EB signal through the metal layer on the SiN film. The metal-coated film consists of 20-nm-thick W and 5-nm-thick Ni layers on a 50-nm-thick SiN film (Figure 1C). The 5-nm-thick Ni layer acts as adhesion between the W layer and the SiN film.

We calculate the scattered electron distribution in a W–Ni-coated SiN film using Monte Carlo (MC) simulation (Figures 1D and E). For 3-kV EB, the irradiated electrons are strongly scattered and completely absorbed in the 20-nm W layer (Figure 1D). At 4 kV, a few electrons reach the water layer (Figure 1E). Therefore, biological specimens under a W–Ni-coated SiN film are subject to very little damage from the irradiated electrons.

**Figure 2:**
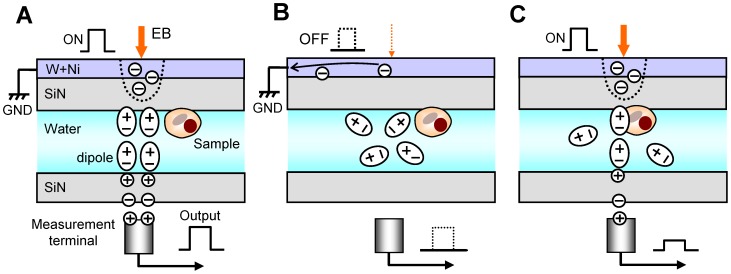
Schematic of our hypothesis of the detection mechanism of the FTE imaging system. (A) If EB irradiates the W–Ni-coated film, the electrons are scattered and absorbed in the film; hence, the negative potential arises in the irradiated position. The negative electric potential influences the electric dipoles of the water molecules and other ions in the sample solution. The negative potential at the irradiated position orientates the water molecules; thereby the negative potential is transmitted to the lower side of the SiN film. (B) When the EB is withdrawn, its negative potential immediately disappears. Therefore, water molecules under the SiN film resort to a random orientation; thereby the detection signal of a measuring terminal is 0 V. Its state is iterated by the square wave of EB. (C) When the EB is irradiated at the biological specimen position, the detection signal is attenuated by the specimens.

Our detection mechanism is shown in Figure 2. During EB irradiation, the electrons are scattered and absorbed by the W layer; hence, the negative electric potential arises in the irradiated position. Its negative potential influences the electric dipoles of the water molecules and other ions in the sample solution (Figure 2A). The negative potential at the irradiated position orientates the water molecules under the SiN film; thereby its negative potential is propagated to the bottom side of the SiN film. Finally, the potential is detected by a measurement terminal under the SiN film through a pre-amplifier. When the EB is turned off, the negative potential at the EB-irradiated position immediately disappears (Figure 2B). Therefore, water molecules under a SiN film adopt a random orientation, and the detection signal disappears. The detection state is iterated by the square wave of EB. When the biological specimens are irradiated with the EB, the detection signal is attenuated by its specimens (Figure 2C).

**Figure 3:**
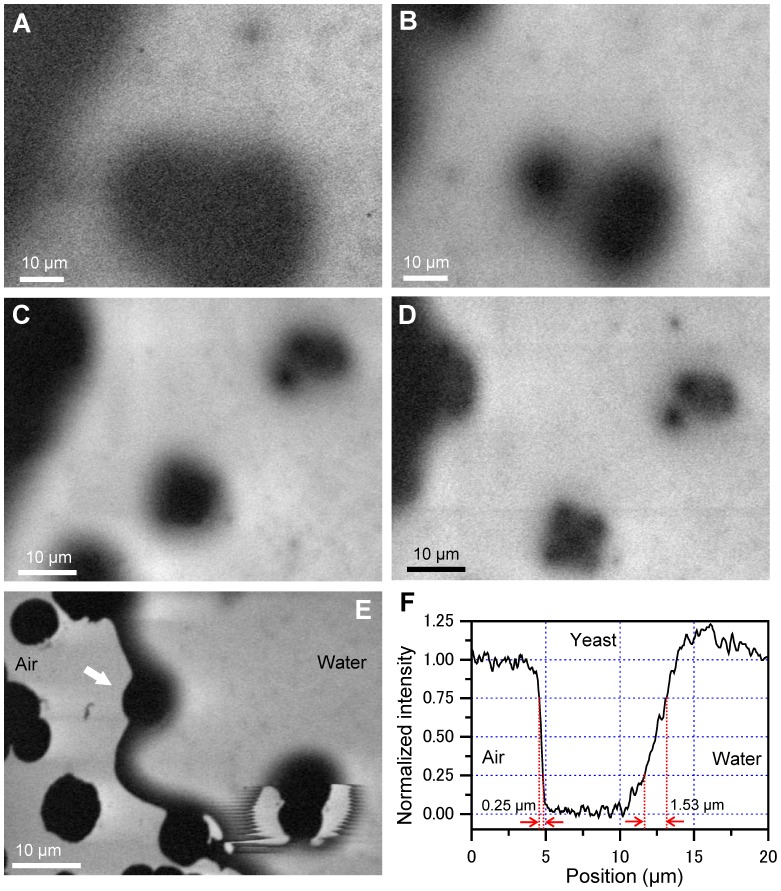
Images of unstained and unfixed yeast in the water. (A) FTE image of yeast obtained under 3-kV EB of 30-kHz chopped frequency and 1500× magnification. In the image, the yeasts show a large ellipsoid with a diameter of 40 μm in clear black contrast. (B) Image of 4-kV EB at 1500× of same area. The yeast image is clearer than that at 3-kV EB. (C) Image of 4-kV EB at 30-kHz frequency and 2000× at another detection area. The image shows two cluster yeasts. (D) Image of the same area of (C) taken at 4-kV and 60-kHz EB. The image clearly shows each yeast in both clusters. (E) A bubble area image, obtained from 4-kV EB of 30-kHz frequency at 2000×. In the image, the left side is the air region and the right side is water. The yeast at the position indicated by the arrow is between air and water. The yeast edge of the air side is sharp, while the water side is broad. (F) Line plot of the centre of the yeast at the white arrow in (E). The falling-edge width under air and water conditions are 0.25 μm and 1.53 μm, respectively. All scale bars are 10 μm.

We first observe the FTE images of unstained yeasts in a water layer sealed in the gap between two SiN films (Figure 3). At an EB of 3 kV and 30-kHz frequency, the yeasts show a large ellipsoid with a diameter of 40 μm of black contrast (Figure 3A). Using 4-kV EB, the FTE image shows two yeasts with diameters of 10 and 20 μm, respectively, and clear contrast (Figure 3B). To further investigate the FTE image, the EB frequency is varied between 30 and 60 kHz (Figures 3C and D). The cluster yeast image is improved at the 60-kHz frequency.

Next, we image the bubble area in the specimens (Figure 3E). In the observation image, the left side is the air region and the right side is water. The yeast highlighted with the white arrow is located at the boundary of air and water. Interestingly, the edge of the yeast at the air side is very sharp. On the other hand, the water side is broad. The difference in the edges is clearly identified from a line plot of the yeast centre (Figure 3F). The falling-edge width under air and water conditions is 0.25 μm and 1.53 μm, respectively, which is defined as the distance over which the normalized intensity decreases from 0.75 to 0.25 in the lineout. These differences may represent the different characteristics of the spread at transmission of the frequency electric signal between air and water. At the yeast of the right-lower side in Figure 3E, the edge between air and water is uneven. Because the electric dipoles of the water molecules are influenced by the negative potential oscillation in the chopped-EB-irradiated position, the water edge is probably pulsed according to its electric-field oscillation.

**Figure 4:**
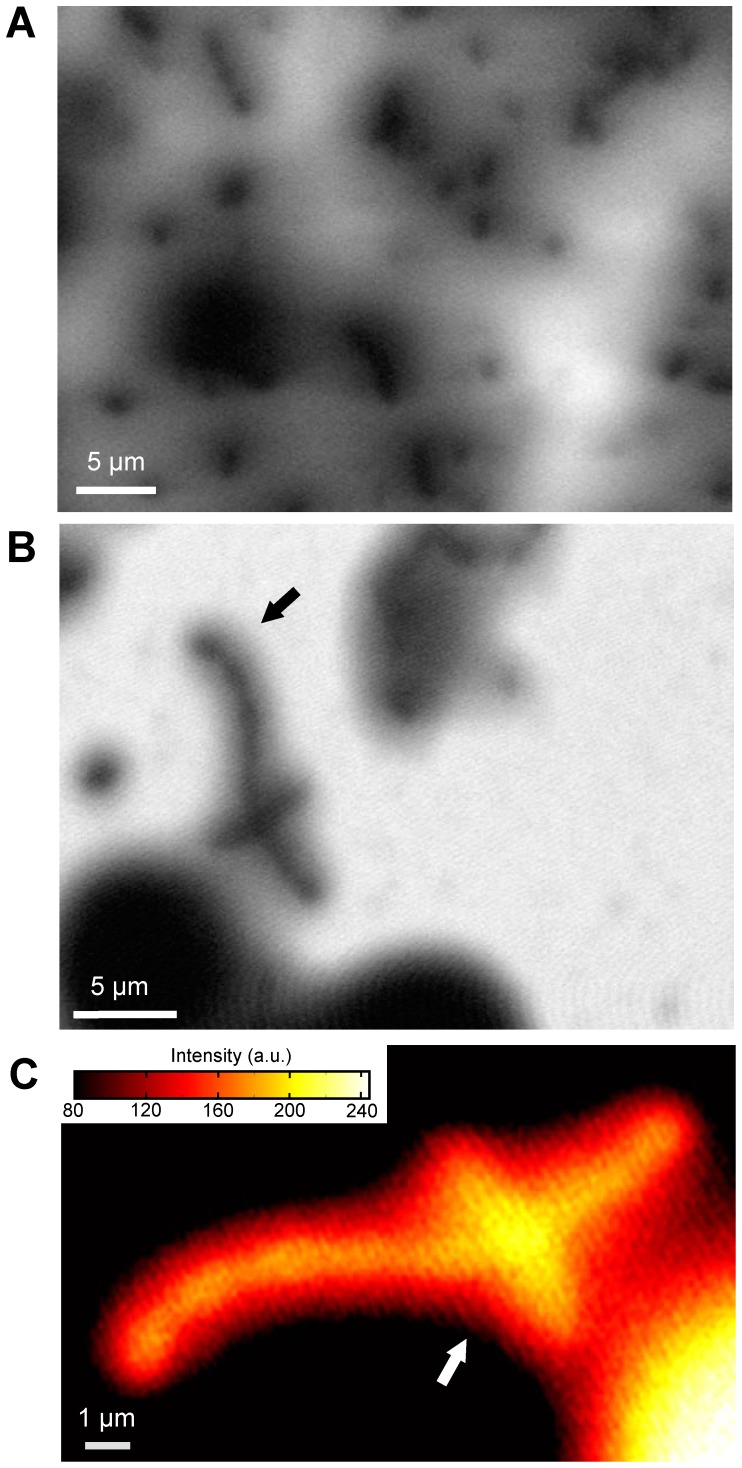
Images of unstained yeast with environmental bacteria in water. (A) At 3-kV EB of 30 kHz with 3000×, the various bacteria of the spherical or cylindrical shapes are scattered around the yeast, with sizes of 1−3 μm. (B) At 4-kV EB with 4000× magnifications, a tubular bacterium was clearly observed. (C) An expanded bacteria image at the reversed intensity of pseudo-colour indicated by the white arrow in (B). The image intensity is increased at the crossover point of two bacteria. Scale bars (A) and (B) 5 μm; (C) 1 μm.

Finally, we measure the yeasts with environmental bacteria (Figure 4). At the 3-kV EB, the various bacteria of the spherical or cylinder shapes are scattered around the yeasts, with sizes of 1−3 μm (Figure 4A). At the 4-kV EB with 4000× magnifications, a tubular bacterium is clearly observed (Figure 4B). To further investigate the bacterium indicated by the black arrow, we create a pseudo-colour in an expanded image (Figure 4C). Some constrictions are visible in the bacterium. Moreover, the image intensity increases at the crossover point of the two bacteria (Figure 4C white arrow). This suggests that the FTE image is similar to the transmission image, depending on the sample volume.

## Discussion

Our FTE imaging method enables observation of unstained biological specimens in water at high contrast. Furthermore, the irradiated electrons are scattered and absorbed in the W–Ni-coated SiN film on the biological specimens; hence, our method is capable of undamaged observation. The irradiated EB is modulated by 30−60 kHz chopping and 3−4 kV acceleration (Figure 1). Therefore, the negative potential oscillation arises at the EB-irradiated position in the W–Ni-coated SiN film, which is transmitted to the bottom SiN film through the biological samples in the liquid layer. Finally, the specimen images are observed by the electric frequency signal from the measurement terminal. A similar electric-field detection method was adopted for the medical imaging of the human body, which is called electrical impedance tomography (EIT) [19]–[23]. Furthermore, the ultrasonic imaging system by SEM (SEAM) was already developed from a focused modulated EB using the deflection plate [24], [25]. In our FTE system, we combined both technologies of EIT and SEAM system using the atmospheric sample holder.

We assume that the mechanism of propagation of the electric frequency signal at the water layer is based on the electric dipoles of the water molecules and various ions (Figure 2). At the negative potential in the W–Ni-coated SiN film by the ON state of EB, the electric dipoles are aligned according to the electric potential. Water has a high electric permittivity; therefore, the electric-potential oscillation in the metal-coated SiN film is propagated to the lower SiN film through the sample solution. On the other hand, the biological specimens consist of amino acids, organic matters, and lipids, with low electric permittivity. Therefore, these specimens decrease the transmission signal of the electric oscillation. The image contrast is influenced by the thickness of the biological specimens, which is similar to the transmission image (Figure 4C). In our method, yeasts with a diameter greater than 10 μm are sealed in the sample holder; therefore, the transmission length of the signal in water is approximately 10 μm. This length is greater than the high-voltage EB of 1 MeV [7], [8]. Furthermore, our system is more convenient and inexpensive compared with high-voltage transmission electron microscopy. In our experiments, the biological specimens were attached to the SiN film. Therefore, its movements were not detected. However, when the specimens moved, its images probably are unclear in a present system under 160-sec scanning time. Therefore, our next system has to improve at faster scanning time to detect movements of the biological specimens.

The spatial resolution of our method is currently unsatisfactory at approximately 200 nm. Therefore, we are working on improving the spatial resolution. To reach 10-nm resolution, we must investigate the physical process of the signal transmission mechanism. In the future, we plan to develop a high-resolution system using high-frequency EB chopping and a 1-GHz detection amplifier. Furthermore, this system will be introduced to high-resolution field-emission SEM. We expect our future FTE system to enable the imaging of unstained biological specimens in water with a resolution greater than 10 nm, allowing observation of living viruses and macromolecular proteins.

In conclusion, we have successfully imaged unstained specimens in water using a newly developed FTE system based on SEM. The specimens in the water layer are placed under the W–Ni-coated SiN film, which is sealed with another 50-nm SiN film using a sample holder. The resulting images clearly show unstained and unfixed yeasts and bacteria in water. Our method offers very low radiation damage to the sample, and the resulting image is similar to the transmission image, depending on the sample volume. Our developed method can easily be utilized for the observation of various biological specimens including living bacteria, viruses, and protein complexes in water. Furthermore, our FTE method can be used for diverse liquid samples across a broad range of scientific fields such as the nanoparticles, organic materials, and catalytic materials.

## Materials and Methods

### Metal coating on the SiN film

A 50-nm-thick SiN film supported by a 0.4 mm × 0.4 mm square window in a Si frame (4 × 4 mm^2^, 0.38-mm thick; Silson Ltd., UK) was coated with W and Ni using a magnetron sputter machine (Model MSP-30T, Vacuum Device Inc., Japan). The Ni sputter conditions were 1.1-Pa Ar pressure, 200-mA current, and 5-sec sputter time. The W sputter conditions were 1.1 Pa, 200 mA, and 20 sec. The distance between the sputter target and SiN films were both 50 mm. The deposited W and Ni layers were 20-nm and 5-nm thick, respectively, as shown in the cross-section image obtained by a field-emission (FE) SEM (JSM-7000F, JEOL, Japan).

### Sample preparation

A yeast sample of *Sacharomyces cerevisiae* was obtained from DCL Yeast Ltd. (UK). Active dried yeast (10 mg) was dissolved in 1 ml of solution containing 0.5% (w/v) trehalose (Hayashibara Inc., Japan) and 0.5% NaCl. To ascertain the presence of yeast, a sample solution was observed by optical microscope (400×, Carl Zeiss Axio Observer A1, Germany) before preparation of the sample holder. For the yeast solution with environmental bacteria, the fresh yeasts solution was preserved at room temperature (23 °C) for one week.

### Atmospheric sample holder

The developed sample holder maintained the sample solution at atmospheric pressure in the sample space between a W–Ni-coated 50-nm SiN film and a 50-nm SiN film (Figure 1). The two SiN films incorporating the liquid sample were sealed using the two sample-holding parts with double-sided tape, and the holding pieces were coupled using two screws. The sample holder consisted of an upper aluminium part and a lower acrylic resin part. The upper part was connected to GND, allowing conduction to the metal layer on the SiN film. The lower part, made of resin, had a high resistivity; hence, measurement at the terminal underside of the holder was insulated from the metal-coated SiN film.

### SEM and FTE imaging system

A beam-blanking unit (Sanyu Electron Co., Japan) consisting of deflection plates was introduced into the thermionic emission SEM (JSM-6390, JEOL, Japan). The unit was controlled by a function generator (WF1974, NF Co., Japan) using a square wave between 0 and 10 V and 30−60 kHz frequency. The atmospheric sample holder was fixed onto an aluminium stage on the upper side of the W–Ni-coated SiN film. The sample holder with the biological specimens in water was mounted onto the sample stage, and its measurement terminal was connected to the pre-amplifier (Figure 1A). The electric frequency signal from the pre-amplifier was entered into a lock-in amplifier (LI5640, NF Co., Japan). The XY scanning signal and the output of lock-in amplifier were recorded using a data recorder (EZ7510, NF Co., Japan). The data files were transferred to a personal computer (Intel Core i7, 2.8 GHz, Windows 7), and the FTE images were calculated using Matlab R2007b (Math Works Inc., USA). The observation conditions of SEM were captured under the following parameters: 1500−4000× magnifications, 1280 × 960 pixels, 160-s scanning time, 7-mm working distance, 3−4 kV accelerating EB, and 300−700 pA current.

### Monte Carlo simulations

Electron trajectories in the W–Ni-coated SiN film were calculated by MC simulation using CASINO version 2.42 [26]. For the W and Ni layers and SiN film, the density was 19.3 g/cm^3^, 8.9 g/cm^3^, and 3.1 g/cm^3^, and the thickness was 20 nm, 5 nm, and 50 nm, respectively. Physical models used for simulation were the same as in our previous study [15]. The simulation parameters were as follows: 1,000,000 electrons, EB accelerating voltages 3−4 kV, and EB spot diameter 10 nm. The simulations were performed on a personal computer (Intel Core i7, 2.8 GHz, Windows 7).

## References

[1] Duckett JG, Ligrone R (1995) The formation of catenate foliar gemmae and the origin of oil bodies in the liverwort *odontoschisma denudatum* (Mart.) Dum. (Jungermanniales) : a light and electron microscope study. Annals of Botany 76: 405–419.

[2] Motta PM, Makabe S, Naguro T, Correr S (1994) Oocyte follicle cells association during development of human ovarian follicle. A study by high resolution scanning and transmission electron microscopy. Arch histol cytol 57: 369–394. 7880591 10.1679/aohc.57.369

[3] Minoura N, Aiba S, Higuchi M, Gotoh Y, Tsukada M, et al (1995) Attachment and growth of fibroblast cells on silk fibroin. Biochem Biophys Res Commun 208: 511–516. 7695601 10.1006/bbrc.1995.1368

[4] Lamed R, Naimark J, Morgenstern E, Bayer EA (1987) Scanning electron microscopic delineation of bacterial surface topology using cationized ferritin. J Microbiol Meth 7: 233–240.

[5] Allan-Wojtas P, Truelstrup Hansen L, Paulson AT (2008) Microstructural studies of probiotic bacteria-loaded alginate microcapsules using standard electron microscopy techniques and anhydrous fixation. LWT Food Sci Technol 41: 101–108.

[6] Richards SR, Turner RJ (1984) A Comparative study of techniques for the examination of biofilms by scanning electron microscopy. Water Res 18: 767–773.

[7] Nagata F, Ishikawa I (1972) Observation of wet biological materials in a high voltage electron microscope. Jpn J Appl Phys 11: 1239–1244.

[8] Parsons DF (1974) Structure of wet specimens in electron microscopy. Science 186: 407–414. 4213401 10.1126/science.186.4162.407

[9] Thiberge S, Nechushtan A, Sprinzak D, Gileadi O, Behar V, et al (2004) Scanning electron microscopy of cells and tissues under fully hydrated conditions. Proc Natl Acad Sci USA 101: 3346–3351. 14988502 10.1073/pnas.0400088101 PMC376183

[10] de Jonge N, Peckys DB, Kremers GJ, Piston DW (2009) Electron microscopy of whole cells in liquid with nanometer resolution. Proc Natl Acad Sci USA 106: 2159–2164. 19164524 10.1073/pnas.0809567106 PMC2650183

[11] Glaeser RM (1971) Limitations to significant information in biological electron microscopy as a result of radiation damage. J Ultrastruct Res 36: 466–482. 5107051 10.1016/s0022-5320(71)80118-1

[12] Henderson R, Glaeser RM (1985) Quantitative analysis of image contrast in electron micrographs of beam-sensitive crystals. Ultramicroscopy 16: 139–150.

[13] Egerton RF, Li P, Malac M (2004) Radiation damage in the TEM and SEM. Micron 35: 399–409. 15120123 10.1016/j.micron.2004.02.003

[14] Inayoshi Y, Minoda H, Arai Y, Nagayama K (2012) Direct observation of biological molecules in liquid by environmental phase-plate transmission electron microscopy. Micron 43: 1091–1098. 22424714 10.1016/j.micron.2012.02.001

[15] Ogura T (2010) Direct observation of unstained wet biological samples by scanning-electron generation X-ray microscopy. Biochem Biophys Res Commun 391: 198–202. 19900411 10.1016/j.bbrc.2009.11.031

[16] Ogura T (2012) Direct observation of the inner structure of unstained atmospheric cells by low-energy electrons. Meas Sci Technol 23: 085402.

[17] Ogura T (2008) A high contrast method of unstained biological samples under a thin carbon film by scanning electron microscopy. Biochem Biophys Res Commun 377: 79–84. 18834858 10.1016/j.bbrc.2008.09.097

[18] Ogura T (2012) High-contrast observation of unstained proteins and viruses by scanning electron microscopy. PloS ONE 7: e46904. 23056522 10.1371/journal.pone.0046904 PMC3466209

[19] Boone K, Lewis AM, Holder DS (1994) Imaging of cortical spreading depression by EIT: implications for localization of epileptic foci. Physiol Meas 15: A189–A198. 8087042 10.1088/0967-3334/15/2a/024

[20] McEwan A, Cusick G, Holder DS (2007) A review of errors in multi-frequency EIT instrumentation. Physiol Meas 28: S197–S215. 17664636 10.1088/0967-3334/28/7/S15

[21] Boverman G, Isaacson D, Saulnier GJ, Newell JC (2009) Methods for compensating for variable electrode contact in EIT. IEEE Trans Biomed Eng 56: 2762–2772. 19628445 10.1109/TBME.2009.2027129 PMC2862904

[22] Kulkarni R, Kao TJ, Boverman G, Isaacson D, Saulnier GJ, et al (2009) A two-layered forward model of tissue for electrical impedance tomography. Physiol Meas 30: S19–S34. 19491444 10.1088/0967-3334/30/6/S02 PMC2722942

[23] Nguyen DT, Jin C, Thiagalingam A, McEwan AL (2012) A review on electrical impedance tomography for pulmonary perfusion imaging. Physiol Meas 33: 695–706. 22532291 10.1088/0967-3334/33/5/695

[24] Brandis E, Rosencwaig A (1980) Thermal-wave microscopy with electron beams. Appl Phys Lett 37: 98–100.

[25] Cargill GS (1980) Ultrasonic imaging in scanning electron microscopy. Nature 286: 691–693.

[26] Drouin D, Couture AR, Joly D, Tastet X, Aimez V, et al (2007) CASINO V2.42 − A fast and easy-to-use modeling tool for scanning electron microscopy and microanalysis users. Scanning 29: 92–101. 17455283 10.1002/sca.20000

